# Isolation, Gastroprotective Effects and Untargeted Metabolomics Analysis of *Lycium Minutifolium* J. Remy (Solanaceae)

**DOI:** 10.3390/foods9050565

**Published:** 2020-05-03

**Authors:** Stephanie Rodriguez, Mariano Walter Pertino, Chantal Arcos, Luana Reichert, Javier Echeverria, Mario Simirgiotis, Jorge Borquez, Alberto Cornejo, Carlos Areche, Beatriz Sepulveda

**Affiliations:** 1 Departamento de Química, Facultad de Ciencias, Universidad de Chile, 8320000 Santiago, Chile; funny.ddrpump@gmail.com (S.R.); areche@uchile.cl (C.A.); 2 Laboratorio de Química de Productos Naturales, Instituto de Química de Recursos Naturales, Universidad de Talca, 3460000 Talca, Chile; mwalter@utalca.cl; 3 Departamento de Ciencias Químicas, Universidad Andres Bello, Campus Viña del Mar, Quillota 980, Viña del Mar, 2531098 Valparaiso, Chile; c.arcoscortez@gmail.com (C.A.); lureichert91@gmail.com (L.R.); 4 Departamento de Ciencias del Ambiente, Facultad de Química y Biología, Universidad de Santiago de Chile, 9170022 Santiago, Chile; javier.echeverriam@usach.cl; 5 Instituto de Farmacia, Facultad de Ciencias, Universidad Austral de Chile, 5090000 Valdivia, Chile; mario.simirgiotis@gmail.com; 6 Departamento de Química, Facultad de Ciencias Básicas, Universidad de Antofagasta, Av Coloso S-N, 1240000 Antofagasta, Chile; jorge.borquez@uantof.cl; 7 Escuela de Tecnología Médica, Facultad de Medicina, Universidad Andres Bello, Sazié 2315, 8370092 Santiago, Chile; alberto.cornejo@unab.cl

**Keywords:** coumarins, *Lycium*, metabolomic, HPLC-MS, orbitrap, secondary metabolites, endemic plants

## Abstract

*Lycium minutifolium* J. Remy (Solanaceae) is commonly used as an infusion in traditional medicine to treat stomach pain, meteorism, intestinal disorders, stomach ailments, and other severe problems including prostate cancer and stomach cancer. From the EtOAc extract of *L. minutifolium* bark five known metabolites were isolated using chromatographic techniques. The gastroprotective effects of the EtOAc fraction and edible infusion extract of the bark were assayed on the hydrochloric acid (HCl)/EtOH induced gastric ulcer model in mice to support the traditional use of the plant. The EtOAc extract and the edible infusion showed gastroprotective effect at dose of 100 mg/kg reducing lesions by 31% and 64%, respectively. The gastroprotective action mechanisms of the edible infusion at a single oral dose of 100 mg/kg were evaluated suggesting that prostaglandins, sulfhydryl groups, and nitric oxide are involved in the mode of gastroprotective action. The UHPLC analysis coupled to high-resolution mass spectrometry of the edible infusion showed the presence of twenty-three compounds. Our results can support the gastroprotective properties of the edible infusion extract, and at least can validate in part, the ethnopharmacological uses of the plant.

## 1. Introduction

Peptic ulcer disease refers to a group of ulcerative disorders that occur only in those parts of the digestive tract exposed to acid and pepsin produced by gastric mucosa. Peptic ulcers are associated with fatal complications such as stomach perforation or stomach bleeding that may cause death [1]. So far, the complete pathogenesis of peptic ulceration is incompletely understood. Peptic ulcer disease is generally thought to be a breakdown in the balance between two opposing forces: the aggressive luminal factors and the gastric mucosal barrier. Hydrochloric acid (HCl) secreted by oxyntic cells and pepsin produced by chief cells in the gastric mucosa are well-known aggressive factors [1]. Among exogenous factors implicated in the pathogenesis of peptic ulcers, ethanol, steroid drugs, smoking, stress, non-steroidal anti-inflammatory drugs, genetic influences, viruses and bacteria such as *Helicobacter pylori* can be mentioned [1]. *H. pylori* has been implicated as the main agent of gastric cancer since it is related to chronic gastritis. In Chile, gastric cancer is a serious public health problem where the highest mortality index is in the regions of Maule, Bio-Bio and La Araucania [2]. An alternative for prevention of this condition is the use of combined strategies for the prevention of peptic ulcers with the use of natural gastroprotective agents.

**Figure 1:**
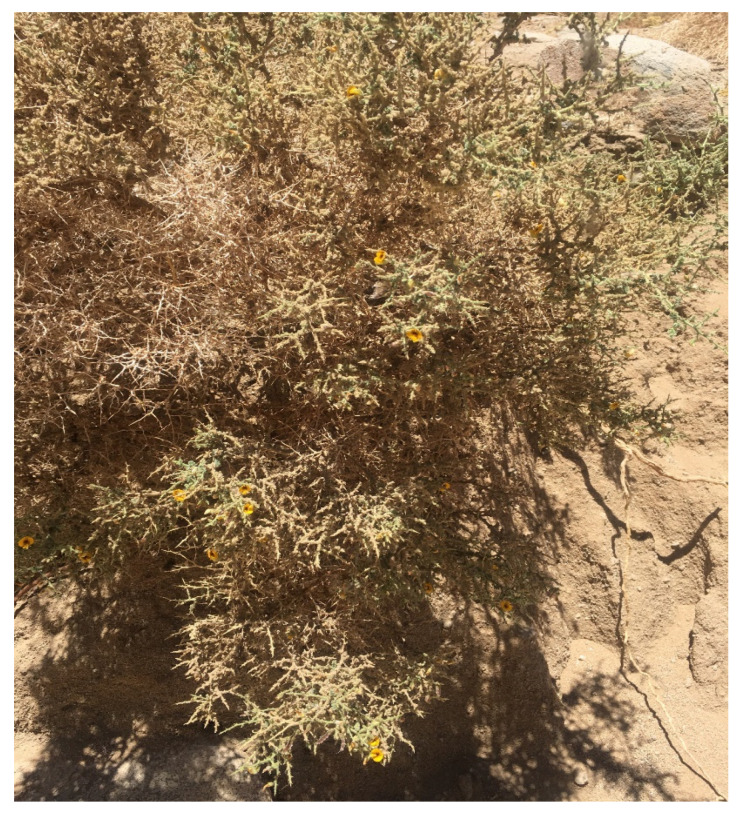
*L. minutifolium* plant.

The plant genus *Lycium* belongs to the Solanaceae family and is distributed in North and South America, Africa and Eurasia. According to Yao et al., 2018 [3], ninety-seven species and six varieties have been recognized: 32 are native to South America, 24 to North America, 24 to Africa and 12 to Eurasia. Among the mentioned species, *Lycium barbarum* L. and *L. chinensis* Mill. produce a famous edible fruit (goji), which is considered as a superfruit with well demonstrated bioactivity properties [3,4,5]. The chemistry of *Lycium* genus cover 355 components from different parts including fruits, root bark, leaves, seeds, and flowers according to Yao et al., 2018 [3]. Among the reported metabolites, coumarins, lignans, phenylpropanoids, flavonoids, amides, alkaloids, anthraquinones, organic acids, terpenoids, sterols, steroids, glycerolipids and peptides can be cited. Pharmacological reports have showed that the edible fruits and rootbarks display antioxidant, antimicrobial, antiaging, antiglaucoma, immunoregulatory, antitumor, hepatoprotective, hypotensive, neuroprotective, spermatogenesis, and blood sugar level-reducing activities, as well in some chronic diseases such as hemoptysis, cough, diabetes, hectic fever and night sweats [3,4,5]. From the point of view of traditional uses, fruits and root bark have been used in traditional Chinese medicine for the treatment of blurry vision, night sweat, fever, kidney deficiency, cough, asthma, diabetes, heart diseases, gynecopathy and neurasthenia [3,4,5]. In relation to *L. minutifolium* J. Remy, studies noticed that this species is endemically distributed in Chile [6], Argentina and Mauritius [3] without no reported use as food or medicine. In Chile, *L. minutifolium* (Figure 1), popularly known as “Caspiche” is endemic and distributed between the Atacama Desert (III Region) and Coquimbo (IV Region). It is commonly used as an infusion in traditional medicine to treat stomach pain, meteorism, intestinal disorders, stomach ailments, heal external wounds and ulcers, and proliferative problems including prostate and stomach cancer [7]. Surprisingly, no chemical or pharmacological studies on secondary metabolites have been performed for *L. minutifolium* so far.

Historically, secondary metabolite studies have been performed through the isolation of components of plants and structural elucidation using spectroscopic and spectrometric data. However, the scenario has changed with the arrival of hyphenated techniques. Liquid chromatography coupled to mass spectrometry (LC-MS) has become a dominant technique for targeted and untargeted metabolomics. Today, it is considered a powerful tool of analysis in food, hospitals, forensic laboratories as well as in basic research. Among LC-MS, high-resolution mass spectrometry has gained much popularity since it is able to discriminate ions based on their small mass difference, helping in detection of isobaric compounds [8].

As part of our studies on Atacama Desert plants, we inform in this work the isolation and structural elucidation of five known compounds plus UHPLC/ESI/MS/MS fingerprints of *L. minutifolium* for the first time. Furthermore, we discuss here the possible mode of gastroprotective action of the edible extract of this plant. 

## 2. Materials and Methods 

### 2.1. Chemicals

TLC (Kieselgel 60 GF254, Merck) was conducted in *n*-hexane/EtOAc or DCM/MeOH mixtures and spots were sprayed with H_2_SO_4_-MeOH (1:9, *v/v*) and heated at 120 °C. Silica gel (Kieselgel 60, Merck 0.063–0.200 mm) and Sephadex (LH-20, Sigma) were used in column chromatography (CC). Technical solvents used in chromatography processes were previously distilled and dried according to standard procedures. 

### 2.2. Plant Material

*Lycium minutifolium* J. Remy were collected in 2017 at “Cuesta el gato” (Copiapo, III Región, Chile) and identified by Prof. Dra Gloria Rojas from the Museo de Historia Natural, Santiago-Chile. A voucher specimen (Nº LM120217) is kept at the Natural Product Lab. of the Universidad de Chile.

### 2.3. Extraction and Isolation

Dried and pulverized bark parts of *L. minutifolium* (1.2 kg) were macerated with ethyl acetate (3 times, 3.0 L, 3 day/extraction). Then, the organic solvent was concentrated under reduced pressure yielding 7.5 g of extract (EA-EXT). This organic extract (7.0 g) was submitted to flash chromatography on silica gel (63–200 μm, 150 g, column length 25 cm, i.d. 10 cm) and eluted with *n*-hexane/EtOAc mixtures (3.0 L each) of increasing polarity (9:1, 7:3, 1:1, 0:1; *v/v*) to give four fractions.

**Figure 2:**
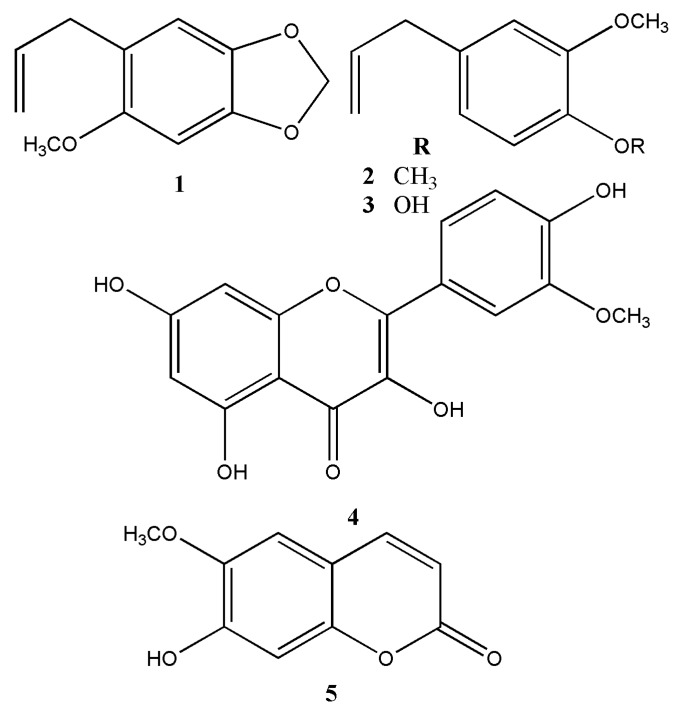
Secondary metabolites isolated from *L. minutifolium*.

Fraction 1 (2.5 g, *n*-hexane/EtOAc 9:1) was chromatographed on a SiO_2_ column (50 g) and eluted with *n*-hexane/EtOAc (1:0, 9:1, 8:2, 7:3, 6:4, 1:1 *v/v*) afforded 210 subfractions (25 mL each). These subfractions were combined based upon TLC monitoring obtaining three main fractions (1A–1C). Repeated CC (silica gel 63–200 μm, 30 g) on fraction 1A (1.0 g) eluted with *n*-hexane/EtOAc mixtures (0–10% EtOAc) led to the isolation of compound **1** (methyleugenol, 25 mg) [9,10] and compound **2** (sarisan, 40 mg) [11] (Figure 2). CC on fraction 1B (0.9 g) eluted with *n*-hexane/EtOAc mixtures (0–25% EtOAc) obtained compound **2** (sarisan, 15 mg) and compound **3** (eugenol, 5 mg) [9,10]. Fraction 1C (0.6 g) was submitted to Sephadex LH-20 (column length 40 cm, i.d. 6.5 cm, MeOH) and then to SiO_2_ CC to afford eugenol **3** (13 mg), and lipids according to ^1^H-NMR. 

Fraction 2 (1.5 g, *n*-hexane/EtOAc 7:3) was permeated on Sephadex LH-20 using MeOH as mobile phase allowing the separation of fatty acids and chlorophylls affording two fractions (2A–2B). Fraction 2A (0.4 g), after repeated CC on silica gel using *n*-hexane/EtOAc mixtures (9:1, 8:2, 7:3, 6:4 and 1:1 *v/v*), produced compound **4** (quercetin-3’-methylether, 2 mg) [5]. Further CC on Fraction 2B (1.1 g) using silica gel afforded compound **5** (scopoletin, 35 mg) [5].

Fraction 3 (2.0 g, *n*-hexane/EtOAc 1:1) was passed on Sephadex LH-20 using MeOH as mobile phase and then was chromatographed on 30 g silica gel with *n*-hexane/EtOAc mixtures (0% to 100%) affording scopoletin **5** and quercetin-3-methylether **4**.

Finally, Fraction 4 (1.0 g, *n*-hexane/EtOAc 0:1) was submitted to CC using Sephadex LH-20 (MeOH) allowing the separation of fats (discarded) and one coumarin according to TLC patron. Repeated CC on silica gel of this fraction afforded scopoletin **5** (20 mg) again (Figure 2).

### 2.4. UHPLC-ESI-MS/MS Studies 

For this study, an infusion was prepared using 3 g of dried chopped bark parts adding deionized water (200 mL) at 100 °C. Then, the solution was lyophilised (Labconco) to obtain 98 mg of edible aqueous extract (EI-EXT).

A Thermo Scientific Dionex Ultimate 3000 UHPLC system, hyphenated with a Thermo high resolution Q-Exactive focus mass spectrometer (Thermo, Bremen, Germany) was used for the analysis. The chromatographic system was coupled to the MS with a Heated Electrospray Ionization Source II (HESI II). XCalibur 3.0 software (Thermo Fisher Scientific, Bremen, Germany) and Trace Finder 3.2 (Thermo Fisher Scientific, San José, CA, USA) were used for UHPLC control and data processing, respectively. Solvent delivery was performed at 1 mL/min using ultra-pure water supplemented with 1% formic acid (A) and acetonitrile with 1% formic acid (B). A program started with 5% B at zero time, then maintained 5% B for 5 min, then changed to 30% B within 10 min, then maintained 30% B for 15 min, then increased to 70% B for 5 min, then maintained 70% B for 10 min, and finally returned to 5% B in 10 min. 

### 2.5. Animals 

Mice weighing 30 ± 3 g were acquired from the Public Health Institute, Santiago, Chile. Standard conditions of Swiss albino mice were reported previously [12].

### 2.6. HCl/EtOH-Induced Lesions in Mice

The gastroprotective activity of the *Lycium minutifolium* extracts was tested at 100 mg/kg on the HCl/EtOH-induced lesion model as described previously [12]. 

### 2.7. HCl/Ethanol-Induced Gastric Lesions in Indomethacin-, NEM- and L-NAME-Pretreated Mice 

To study the involvement of prostaglandins, sulfhydryl compounds, endogenous nitric oxide and vanilloid receptor in the gastroprotective activity of EI-EXT, Indomethacin s.c. (30 mg/kg), NEM s.c. (10 mg/kg), L-NAME i.p. (70 mg/kg) and RR s.c. (3.5 mg/kg) were injected 30 min before the administration of EI-EXT or vehicle as published previously [12,13]. 

### 2.8. Statistical Analysis

Our results were expressed as the mean ± S.D. Statistical differences between treatments and control were performed by one-way analysis of variance (ANOVA) followed by Dunnett’s test. All statistical analyses were performed using the software GraphPad Prism 6 for Windows.

## 3. Results

### 3.1. Isolation of Secondary Metabolites

The bark of *L. minutifolium* was macerated with EtOAc for nine days at room temperature. The crude organic extract obtained after evaporation of the organic solvent was fractionated by flash column chromatography (SiO_2_) yielding four fractions. Each fraction was subjected to repeated permeation with Sephadex LH-20 and chromatography over silica gel to yield five compounds (**1**-**5**). All compounds were isolated and identified using ^1^H-NMR spectroscopic and HR-MS spectrometric techniques and belong to three different classes of compounds: phenylpropanes, flavonoids and coumarins. Among them sarisan **1**, methyleugenol **2**, eugenol **3**, quercetin-3’-methyl ether (isorhamnetin, **4**), and scopoletin **5** are reported for the first time in this species (Figure 2). 

### 3.2. Metabolomic Profiling of the Infusion by Using UHPLC-ESI-MS/MS 

**Figure 3:**
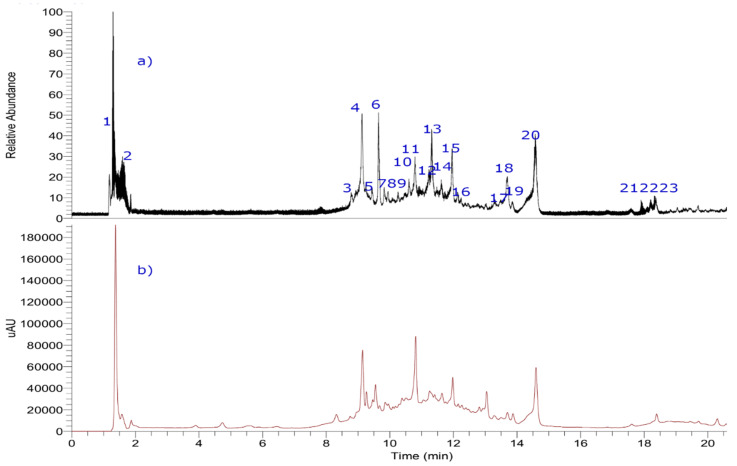
UHPLC-MS chromatograms of *Lycium minutifolium.* (**a**) TIC (total ion current) (**b**) UV at 280 nm.

Ten milligrams of the edible *L. minutifolium* aqueous extract (EI-EXT) was dissolved in fresh water, filtered and injected in the UHPLC-MS/MS machine (Figure 3 and Table 1). This infusion was chosen for metabolomic profiling due to the higher biological activity previously shown in comparison to that of ethyl acetate extract (EA-EXT) on the HCl/EtOH-induced gastric lesions model in mice.

Organic acids: Peak **1** with a molecular anion at *m/z* 191.0557 was identified as quinic acid (C_7_H_11_O_6_^−^) [14,15], while peak **2** was identified as citric acid (*m/z* 191.0199; C_6_H_7_O_7_^−^) [16]. Quinic acid is a cyclic polyol common in plants such as coffee, Tara, Eucalyptus, Urtica, and cinchona. Citric acid is common in citrus fruits and is used as flavoring and chelating agent. 

**Figure 4:**
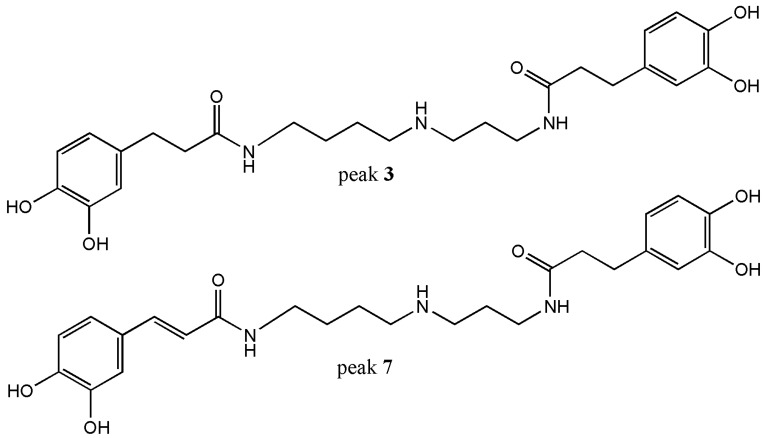
Spermine-type alkaloids detected by UHPLC/ESI/MS/MS from *L. minutifolium*.

Spermine alkaloids: Peak **3** presented a molecular anion at *m/z* 472.2447 and was identified as *N*^1^, *N*^3^-bis-dihydrocaffeoylspermidine (C_25_H_34_N_3_O_6_^−^) and UV absorbance at dihydro caffeoyl structure (287 nm) (Figure 4). This structure was supported by the presence of two diagnostic fragments at *m/z* 308.1976 and 163.0393 [17]. Peak **3** was reported as constituent of *Scopolia tangutica* and *Iochroma cyaneum*, both belonging to Solanaceae family [18,19]. Peak **6** with a molecular ion at *m/z* 472.2453 was considered as a potential unknown with MS/MS fragmentation data closely related to a spermidine derivative such as the peak **3** and peak **7** (see Figure S1 in Supplementary Material). This peak showed two daughter fragments at *m/z* 308.1975 and 163.0392 considered typical daughter fragments for spermine alkaloids like the peak **3**. Peak **6** could be considered a new compound awaiting to be isolated. Peak **7** is a dehydrogenated derivative of peak **3** whose fragments were at *m/z* 334.1769; 308.1977; 306.1820 and 135.0443. Based on these data peak **7** was identified as *N*^1^-caffeoyl-*N*^3^-dihydrocaffeoylspermidine (C_25_H_33_N_3_O_6_^−^). Peak **7** has been reported as metabolite in *Lycium barbarum* [3,4,5,19].

Phenolic acids: Peak **4** was identified as chlorogenic acid (5-caffeoylquinic acid) [14,15]. Peak **5** as the isomer 4-caffeyolquinic acid, and peak **8** as the isomer 3-caffeyolquinic acid. Chlorogenic acid is the main phenolic compound in coffee, artichoke, carrot, kiwi fruit, pears, eggplant, peaches showing anti-inflammatory, antidiabetic, antioxidant, antibacterial, anti-obesity, cardioprotective, hepatoprotective, antipyretic, neuroprotective, antiviral, anti-hypertension and anti-microbial activities [20]. 

Flavonoids: Peak **10** was assigned as quercetin-3-*O*-hexoside-7-*O*-dihexoside based on its molecular ion (*m/z*: 787.1928) and their diagnostic fragments. Peak **11** with a molecular ion at *m/z*: 609.1440 was identified as rutin. Peaks **12**–**14**, **16**–**19** and **23** were assigned to kaempferol-3-*O*-hexoside-pentoside, isorhamnetin-hexoside-rhamnoside, kaempferol-3-*O*-hexoside, eriodictyol, kaempferol or luteolin, quercetin, isorhamnetin and methylisorhamnetin based on HR-MS and their respective diagnostic daughter fragments. All these flavonoids have been reported in *Lycium barbarum* and *L. chinensis* [3,4,5]. 

Coumarins: Only esculin was detected in *Lycium minutifolium* bark based on molecular ion at *m/z*: 339.0714 and their MS^2^ fragment at *m/z*: 177.0190 (sculetin). This peak **15** has been reported as a constituent of *Lycium barbarum* [5].

Tropane alkaloids: Peak **9** was tentatively identified as an atropine derivative based on a diagnostic fragment at 149.0601 with a loss of atropine moiety. From *Lycium europaeum* L. fruits atropine, hyoscyamine and scopolamine have been identified. The presence of these alkaloids could question the safety of *Lycium* fruit for human consumption [5].

Fatty acids: Peaks **20**–**22** were tentatively identified as trihydroxyoleic acid and their isomers (C_18_H_33_O_5_). Remarkably, the presence of oleic acid derivatives was found in *Lycium barbarum* fruits [5].

### 3.3. Gastroprotective Activity

The effects of the EtOAc extract (EA-EXT) and edible infusion (EI-EXT) in HCl/EtOH induced gastric lesion model in mice are shown in Table 2. The EA-EXT inhibited the gastric lesions by 31% while the EI-EXT by 64% at the dose of 100 mg/kg compared with control group. This dose is valid to show the ethnopharmacological properties of these extracts. In addition, we evaluated the possible mode of gastroprotective action of EI-EXT at a single oral dose of 100 mg/kg. The results of EI-EXT on the gastric lesions induced by HCl/EtOH in mice pretreated with Indometacin (IND, 10 mg/kg, s.c.), N-ethylmaleimide (NEM, 10 mg/kg, s.c.), NG-nitro-L-arginine methyl ester (L-NAME, 70 mg/kg, i.p.), or ruthenium red (RR, 3.5 mg/kg, s.c.) are shown in Table 3.

Prostaglandins (PGs) are believed, through PGs production, to be involved in the protection of the stomach mucosa against chemical agents such as ethanol, HCl, and NaOH [21,22]. In our study, pre-treatment with indomethacin (an PGs inhibitor) reduced the gastric protection of EI-EXT (Table 3). This result indicates that PGs are involved in the gastroprotective effect of EI-EXT.

The decrease of sulfhydryl (SH) groups have a relationship with gastric lesions induced by ethanol. Glutathione, a known endogenous sulfhydryl, preserves the integrity of the cell and acts as a scavenger of free radicals, an antioxidant, and maintains the immune system as well as protein synthesis and their surface [23]. In our study, pre-treatment with *N*-ethylmaleimide (NEM, SH-blocker) reduced the protection showed by EI-EXT. This evidence implies that endogenous SHs participate in the gastroprotective activity of this edible extract.

Nitric oxide (NO) is implied in gastric mucosa defense through the regulation of the gastric blood flow, angiogenesis and mucus secretion [24]. In our study, pre-treatment with N^G^-nitro-L-arginine methyl ester (L-NAME, an inhibitor of NO synthase) reduced the gastroprotective activity of EI-EXT, suggesting that the protective effect of this edible extract is through the participation of endogenous NO (Table 3).

Capsaicin-sensitive sensory neurons through vanilloid receptors on the gastric mucosa protect via the regulation of acid secretion, gastric motility, gastric blood flow and mucus production [1]. In this study, pre-treatment with RR did not produce significant changes in the protection of EI-EXT suggesting that this extract did not imply VR. 

Nature has provided many gastroprotective drugs during the last decades including carbenoxolone from *Glycyrrhiza glabra*, solon from sophoradin and gefarnate from cabbage (*Brassica oleracea* L.) as the most important gastroprotective agents [23]. So many crude drugs have been reported as gastroprotective agents and this information is summarized in some excellent reviews by Tundis et al., 2008 [25], Mota et al., 2009 [26], Sumbul et al., 2011 [27] and Khan et al., 2018 [28]. Regarding bioactive compounds, a polyssacharide fraction from traditional plant *Handroanthus heptaphyllus* constituted by arabinogalactans II has showed gastroprotective activity at 10 mg/kg on gastric lesions induced by ethanol. Moreover, this fraction at the same doses presented gastric ulcer healing in rats through the inhibition of mucus and GSH depletion. In another study, a polysaccharide fraction at 100 mg/kg from the medicinal plant *Bletilla striata* displayed a reduction in the formation of gastric lesions by the increase in PGE2 content, mitigating of oxidative stress, suppression of MAPK/NF-kB signaling pathway in gastric tissue and a reduction in the levels of pro-inflammatory agents as TNF-α, IL-1β, IL-6 and IL-18 [29]. Arunachalam et al., 2019 [30] reported that a hydroethanolic extract of *Cochlospermum regium* at 100 mg/kg showed preventive and curative activity in different gastric ulcer models supporting the traditional use of this plant. In the same study, it was demonstrated that the mechanism of gastroprotective action and antiulcer activity implied an increase in mucus production, gastric secretion inhibition, activation of K^+^_ATP_ channels and α-2-adrenergic receptor, augmentation of antioxidant activity and stimulation of PGs and NO synthesis. In the case of *Lycium* species referred to gastroprotective and ulcerogenic activities, only *Lycium chinense* has been reported to have these properties [31,32]. An ethyl acetate extract of the aerial part of *L. chinense* showed gastroprotective activity based on its antioxidant, anti-inflammatory, anti-secretory and anti-apoptotic effects [32]. Pretreatment with ethyl acetate extract at 50–400 mg/kg attenuated the ethanol-induced gastric lesions either increasing oxidative stress markers (GSH and SOD), pH of gastric juice, and mucin soluble content, or decreasing MOP activity, MDA elevation, caspase-3-expresion and inflammatory markers (such as TNF-α, IL-1β, iNOS and COX-2). In a similar way, Cheng et al. 2016 [31] reported gastroprotective properties at doses of 50, 100, 200 and 400 mg/kg of the root bark of *L. chinense* suggesting that the mechanisms involved could be related to the scavenging of free radicals, antioxidant, anti-inflammatory and pro-inflammatory marker regulations. *Lycium barbarum,* a closely related plant to *Lycium chinense,* displayed anti-ulcer activity against water-immersion restraint stress, acetic acid and pylorus-ligation models in rats and this activity was related to the presence of polysaccharides isolated from water extract [33]. In our study, we have demonstrated that edible infusion (100 mg/kg) of *L. minutifolium* possesses gastroprotective properties against HCl/EtOH induced gastric lesion model in mice suggesting that this protective effect is through the participation of endogenous NO, endogenous SHs, and prostaglandins. 

On the other hand, the mortality after peptic ulcer disease decreased significantly in the last few decades, due to treatment of *H. pylori* infections [34]. Several bioactive compounds were key defensive factors against this bacteria such as: arabinogalactanes (jambo, mangoes, and goji Lycium fruits), other polysaccharides obtained from fruits (*Maytenus ilicifolia* Mart. ex Reissek) and acidic heteroxylans, (for instance from Olea fruits), rhamnogalacturonanes (ubiquitous in several grapes and ginseng roots) and terpenoids, (such as pinene, lupeol, limonene, citral, nomiline and ursolic acid), and flavonoids such as flavonol derivatives have been showed to display this activity [28,35]. These compounds can prevent the invasion, colonization, and adherence of *H. pylori* into the cells of the stomach and prevent gastric cancer, suppressing cancer growth, which is very common in *H. pylori*-infected patients.

The potential bioactivity of this plant could be attributable to the chemical diversity found in the edible infusion such as phenolic acids, alkaloids and flavonoids detected by UHPLC/ESI/MS/MS. These compounds are believed to play an essential role in these bioassays as indicated in our study and others [26,27,36,37]. Further studies are required to isolate and evaluate the gastroprotection of all pure compounds. 

## 4. Conclusions

In the present study, we isolated five known compounds named as methyl eugenol, sarisan, eugenol, quercetin-3’-methylether (isorhamnetin), and scopoletin for the first time from the ethyl acetate extract of *Lycium minutifolium*. Then, an edible infusion extract was prepared, and both extracts at 100 mg/kg displayed gastroprotective activity (31% and 64%) on the HCl/EtOH-induced gastric lesion model in mice. Our findings suggest that prostaglandins, sulfhydryl groups, and nitric oxide are involved in the mode of gastroprotective action of this edible extract. Finally, in the edible extract twenty-three compounds were tentatively detected by UHPLC/ESI/MS/MS including diverse compounds such as organic acids, spermine and tropane alkaloids, phenolic acids, flavonoids and fatty acids. Furthermore, peak **6** is considered an unknown spermine-type alkaloid. These results based on ethnomedicinal properties provide the basis for the utilization of *L. minutifolium* as a source of potential compounds or mixtures for the prevention of gastric ulcers. 

## Figures and Tables

**Table 1:** UHPLC-PDA-MS orbitrap mass spectral data of lyophilized infusion of *Lycium minutifolium.*

Peak	T_R_ (min.)	Tentative Identification	[M-H]^−^	Theoretical Mass (*m/z*)	Measured Mass (*m/z*)	Accuracy (ppm)	MS^n^ Ions (ppm)
1	1.35	Quinic acid	C_7_H_11_O_6_^−^	191.0561	191.0557	−1.93	109.0286
2	1.87	Citric acid	C_6_H_7_O_7_^−^	191.0192	191.0199	3.36	111.0080
3	8.79	*N*1,*N*3-bis dihydrocaffeoyl spermidine	C_25_H_34_N_3_O_6_^−^	472.2455	472.2447	−0.47	308.1976; 163.0393
4	9.13	Chlorogenic acid (5-Caffeoylquinic acid)	C_16_H_17_O_9_^−^	353.0876	353.0879	3.52	191.0557; 707.1813 (2M-H adduct)
5	9.24	Chlorogenic acid (4-Caffeoylquinic acid)	C_16_H_17_O_9_^−^	353.0876	353.0879	3.52	191.0557; 707.1813 (2M-H adduct)
6	9.65	bis dihydrocaffeoyl spermidine derivative	C_25_H_34_N_3_O_6_^−^	472.2455	472.2453	−0.47	308.1975; 163.0392
7	9.82	*N*1,caffeoyl-*N*3-dihydrocaffeoyl spermidine	C_25_H_33_N_3_O_6_^−^	470.2299	470.2293	1.11	334.1769; 308.1977; 306.1820; 135.0443
8	9.94	Chlorogenic acid (3-Caffeoylquinic acid)	C_16_H_17_O_9_^−^	353.0878	353.0881	0.90	191.0555
9	10.09	Atropine derivative	C_17_H_18_NO_5_^−^	316.1188	316.1192	−1.4	149.0601
10	10.25	Quercetin-3-O-hexoside-7-O-hexoside-hexoside	C_33_H_39_O_22_^−^	787.1938	787.1928	2.03	609.1457; 301.0341
11	10.78	Rutin	C_27_H_29_O_16_^−^	609.1455	609.1440	−2.8	301.0342; 300.0269; 179.0432
12	11.23	Kaempferol-3-O-hexoside-pentoside	C_27_H_29_O_15_^−^	593.1511	593.1501	1.62	285.0405; 255.0279
13	11.49	Isorhamnetin-hexoside-rhamnoside	C_28_H_31_O_16_^−^	623.1616	623.1611	1.28	477.1014; 315.0499; 300.0264
14	11.32	Kaempferol-3-O-hexoside	C_21_H_19_O_11_^−^	447.0933	447.0919	1.28	285.0401
15	11.63	esculin	C_15_H_15_O_9_^−^	339.0722	339.0714	0.46	177.0190
16	13.50	Eriodictyol	C_15_H_11_O_6_^−^	287.0556	287.0550	2.15	135.0442
17	13.67	Kaempferol or luteolin	C_15_H_9_O_6_^−^	285.0401	285.0393	2.90	179.0432; 151.0029
18	13.84	Quercetin	C_18_H_15_O_7_^−^	301.0342	301.0351	3.01	151.0034
19	14.57	Isorhamnetin	C_16_H_11_O_7_^−^	315.0510	315.0506	2.41	300.0273
20	17.92	Trihydroxyoleic acid	C_18_H_33_O_5_^−^	329.2333	329.2322	2.41	-
21	17.92	Trihydroxyoleic acid	C_18_H_33_O_5_^−^	329.2333	329.2321	2.23	-
22	18.19	Trihydroxyoleic acid	C_18_H_33_O_5_^−^	329.2333	329.2322	2.46	-
23	18.33	Methyl isorhamnetin	C_17_H_13_O_7_^−^	329.0665	329.0655	2.97	271.0243

**Table 2:** Gastroprotective activity of the EA-EXT (organic extract) and EI-EXT (edible extract) on hydrochloric acid (HCl)/EtOH-induced gastric lesions in mice.

Treatment	*n*	Lesion Index (mm)	% Lesion Reduction	Dose (mg/Kg)
EA-EXT	7	31.3 ± 3.2 **	31 *	100
EI-EXT	7	16.2 ± 3.7	64 *	100
Lansoprazole	7	14.7 ± 4.8	69 *	30
Control	7	45.4 ± 4.5	-	-

The results were expressed as mean ± SD * *p* < 0.01; significantly different compared with the control and ** *p* < 0.01 significantly different compared with lansoprazole (analysis of variance (ANOVA) followed by Dunnett’s test). *n* = number of mice.

**Table 3:** Protective effect of edible infusion (EI-EXT) on the HCl/EtOH model in indomethacin-, NEM-, L-NAME, and RR-pretreated mice.

Treatment	Dose (mg/kg)	Lesion Index (mm)
Control	-	45.4 ± 4.5
**EI-EXT**	100	16.2 ± 3.7 *
IND + **EI-EXT**	10 + 100	39.8 ± 5.2
NEM + **EI-EXT**	10 + 100	36.1 ± 5.5
L-NAME + **EI-EXT**	70 + 100	40.0 ± 5.8 *
RR + **EI-EXT**	3.5 + 100	18.1 ± 3.5
Carbenoxolone	100	14.6 ± 4.2 *

Results were expressed as mean ± SD, *n* = 7. Analysis of variance followed by Dunnett’s test. * *p* < 0.01 compared with the respective control.

## Supplementary Materials

The following are available online at https://www.mdpi.com/2304-8158/9/5/565/s1, Figure S1: Full HR-orbitrap MS spectra and structures of some representative compounds, peaks 3, 4, 6, 7, 11 and 19.
