# Supplementary material for: Isolation, Gastroprotective Effects and Untargeted Metabolomics Analysis of Lycium Minutifolium J. Remy (Solanaceae)
Source: Foods. 2020 May 3;9(5):565. doi: 10.3390/foods9050565 (PMC7278853; doi:10.3390/foods9050565)
Supplement: Supplementary file 1 [file foods-09-00565-s001.pdf]

Article

Isolation, Gastroprotective Effects and Untargeted Metabolomics Analysis of *Lycium minutifolium* J. Remy (Solanaceae)

Stephanie Rodriguez[1], Mariano Walter Pertino[2], Chantal Arcos[3], Luana Reichert[3], Javier Echeverria[4], Mario Simirgiotis[5], Jorge Borquez[6], Alberto Cornejo[7], Carlos Areche[1], Beatriz Sepulveda[3]*

[1]Departamento de Química, Facultad de Ciencias, Universidad de Chile, Santiago, Chile

[2]Laboratorio de Química de Productos Naturales, Instituto de Química de Recursos Naturales, Universidad de Talca, 3460000 Talca, Chile

[3]Departamento de Ciencias Químicas, Universidad Andres Bello, Campus Viña del Mar, Quillota 980, Viña del Mar, Chile

[4]Departamento de Ciencias del Ambiente, Facultad de Química y Biología, Universidad de Santiago de Chile, Santiago, Chile.

[5]Instituto de Farmacia, Facultad de Ciencias, Universidad Austral de Chile, Valdivia, Chile

[6]Departamento de Química, Facultad de Ciencias Básicas, Universidad de Antofagasta, Av Coloso S-N, Antofagasta, Chile

[7]Escuela de Tecnología Médica, Facultad de Medicina, Universidad Andres Bello, Sazié 2315, 8370092, Santiago-Chile.

* **Correspondence:** Beatriz Sepulveda; bsepulveda@uc.cl (B.S.); Tel.: +56-063-2244369

Figure S1. (a-f): Full HR-orbitrap MS spectra and structures of some representative compounds, peaks 3, 4, 6, 7, 11 and 19.

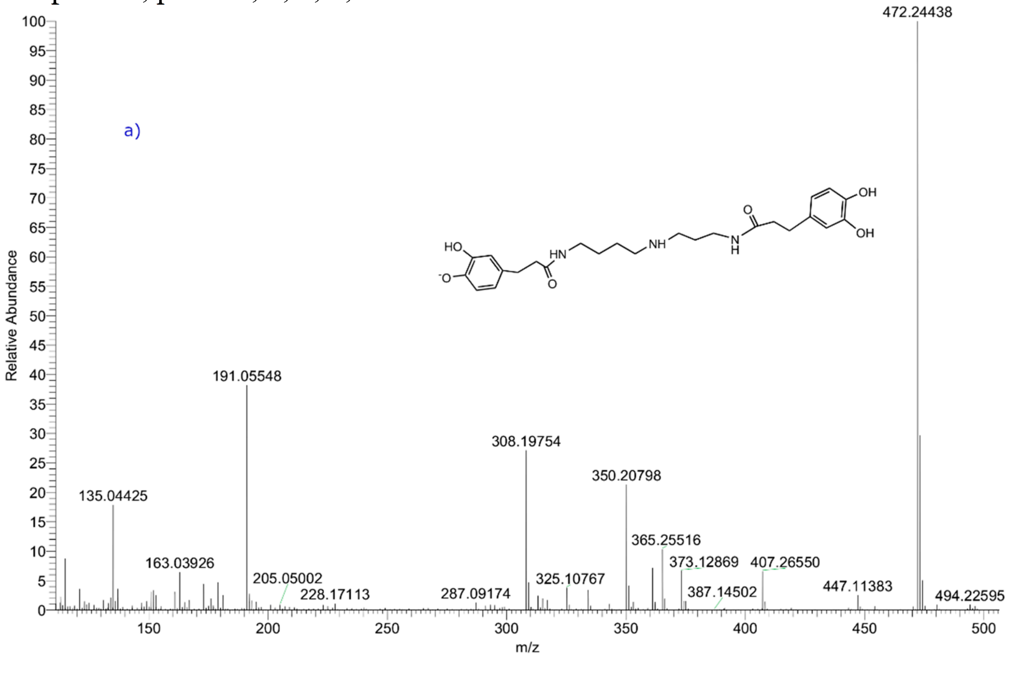

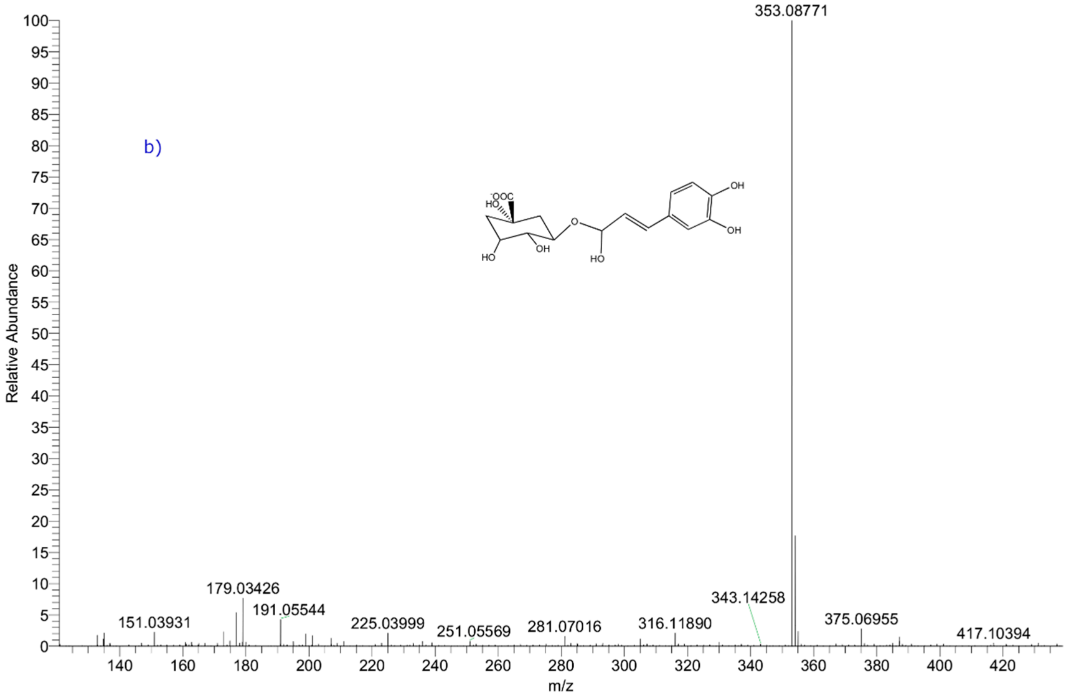
b)
353.08771
179.03426
191.05544
151.03931
225.03999
251.05569
281.07016
316.11890
343.14258
375.06955
417.10394
Relative Abundance
m/z
HO
OH

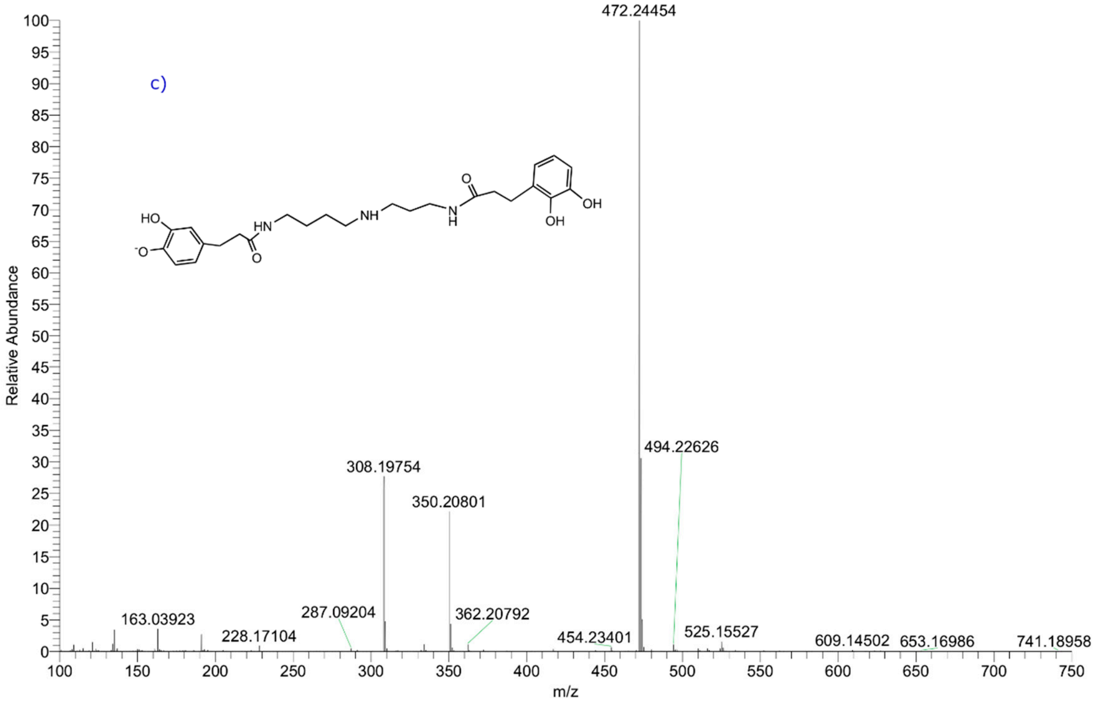
c)
472.24454
494.22626
308.19754
350.20801
163.03923
228.17104
287.09204
362.20792
454.23401
525.15527
609.14502
653.16986
741.18958
Relative Abundance
m/z
HO
HN
NH
OH

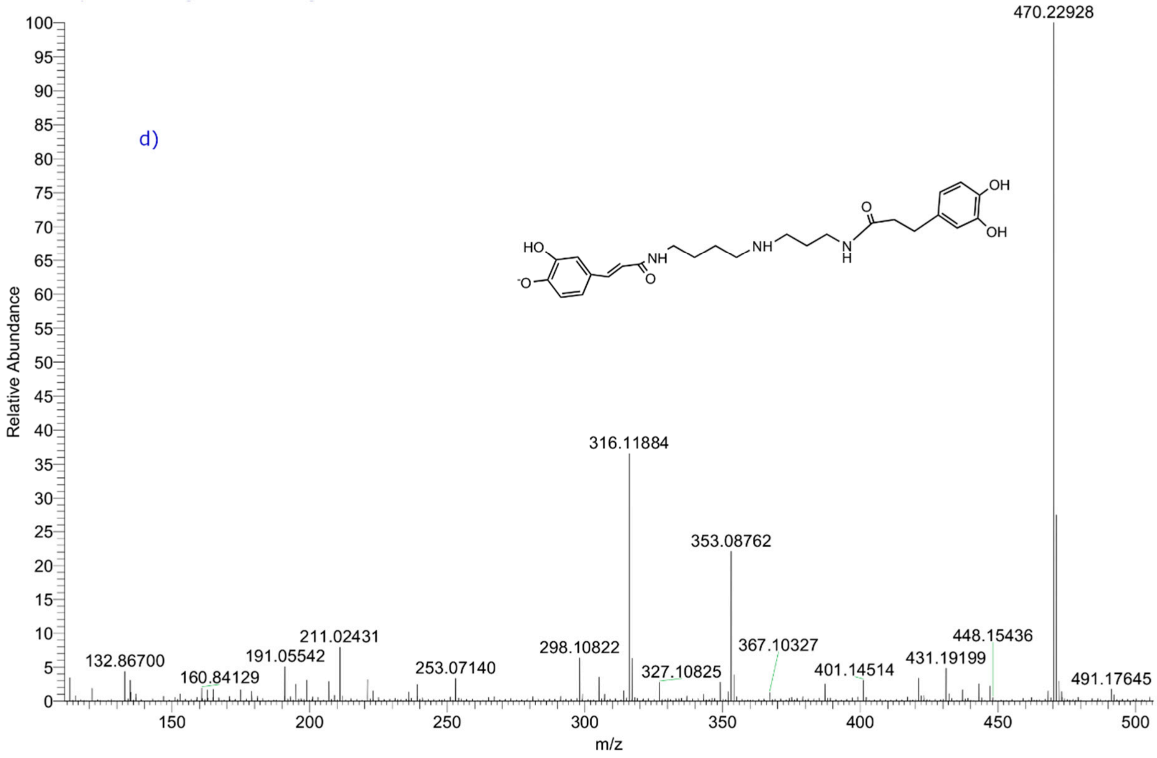
d)
Relative Abundance
m/z
470.22928
316.11884
353.08762
132.86700
160.84129
191.05542
211.02431
253.07140
298.10822
327.10825
367.10327
401.14514
431.19199
448.15436
491.17645

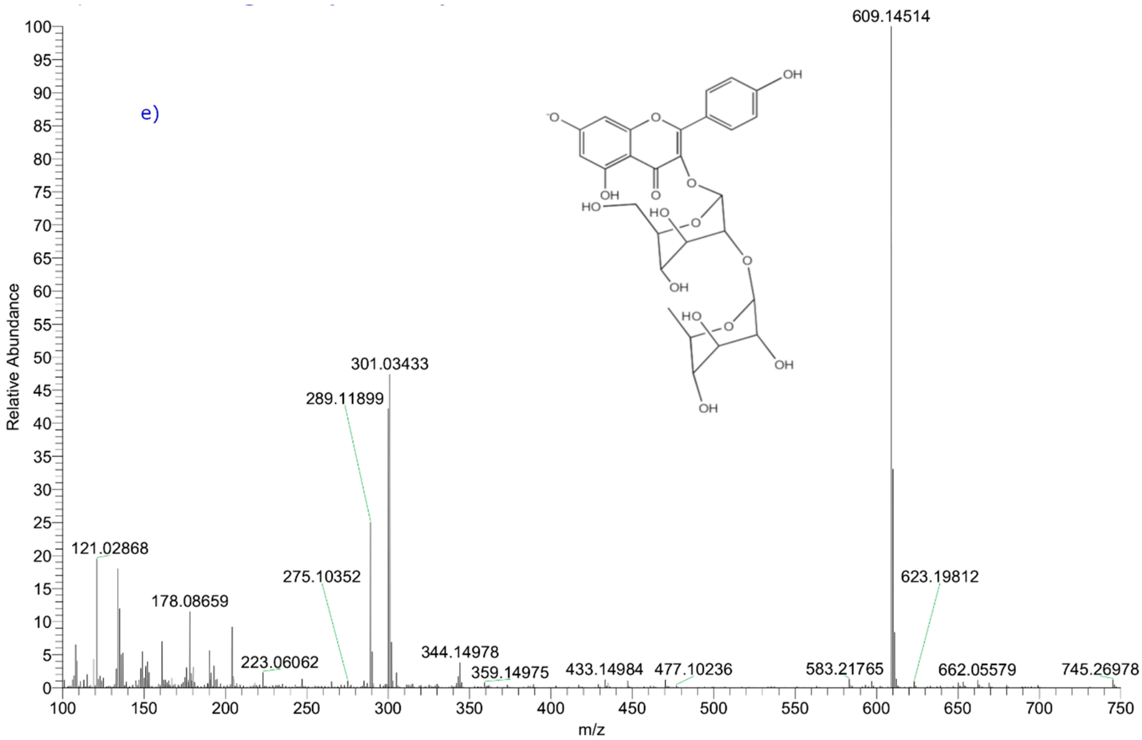
e)
Relative Abundance
m/z
609.14514
301.03433
289.11899
121.02868
178.08659
275.10352
223.06062
344.14978
359.14975
433.14984
477.10236
583.21765
623.19812
662.05579
745.26978

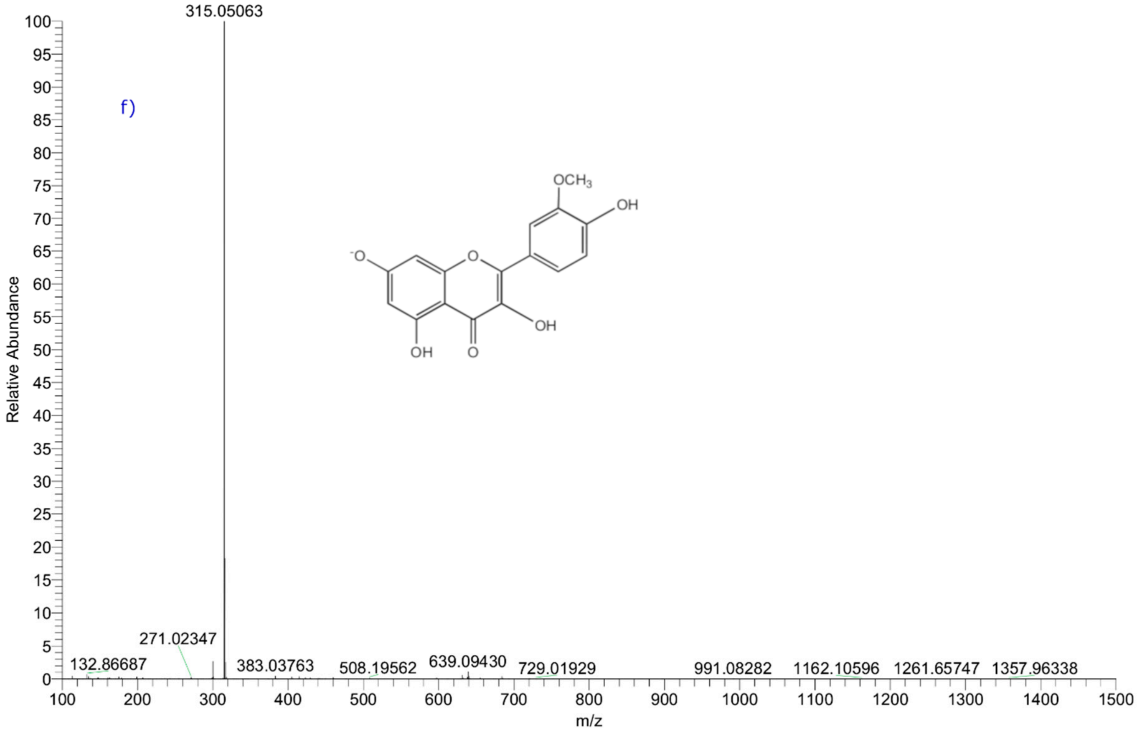
315.05063
f)
OCH3
OH
-O
O
OH
OH
O
Relative Abundance
271.02347
132.86687
383.03763
508.19562
639.09430
729.01929
991.08282
1162.10596
1261.65747
1357.96338
m/z
